# Improving the Representativeness of Behavioral and Clinical Surveillance for Persons with HIV in the United States: The Rationale for Developing a Population-Based Approach

**DOI:** 10.1371/journal.pone.0000550

**Published:** 2007-06-20

**Authors:** A. D. McNaghten, Mitchell I. Wolfe, Ida Onorato, Allyn K. Nakashima, Ronald O. Valdiserri, Eve Mokotoff, Raul A. Romaguera, Alice Kroliczak, Robert S. Janssen, Patrick S. Sullivan

**Affiliations:** 1 Centers for Disease Control and Prevention, Atlanta, Georgia, United States of America; 2 Michigan Department of Community Health, Detroit, Michigan, United States of America; 3 Health Resources and Services Administration, Rockville, Maryland, United States of America; University of California, San Francisco, United States of America

## Abstract

The need for a new surveillance approach to understand the clinical outcomes and behaviors of people in care for HIV evolved from the new challenges for monitoring clinical outcomes in the HAART era, the impact of the epidemic on an increasing number of areas in the US, and the need for representative data to describe the epidemic and related resource utilization and needs. The Institute of Medicine recommended that the Centers for Disease Control and Prevention and the Heath Resources and Services Administration coordinate efforts to survey a random sample of HIV-infected persons in care, in order to more accurately measure the need for prevention and care services. The Medical Monitoring Project (MMP) was created to meet these needs. This manuscript describes the evolution and design of MMP, a new nationally representative clinical outcomes and behavioral surveillance system, and describes how MMP data will be used locally and nationally to identify care and treatment utilization needs, and to plan for prevention interventions and services.

## Introduction

HIV/AIDS surveillance programs in all US states collect a core set of information about persons diagnosed with, living with and dying from HIV infection and AIDS [1]. Supplemental surveillance projects have historically provided complementary information about clinical outcomes of HIV infection and behaviors of HIV-infected persons. Although these supplemental surveillance activities have been instrumental in providing additional information for describing the epidemic, the utility of these surveillance projects, which were started in 1990, has become progressively limited over time.

The Medical Monitoring Project (MMP) arose out of a need for a nationally representative, population-based surveillance system to assess behaviors, clinical outcomes, and the quality of care for persons with HIV infection who are receiving care. MMP is a surveillance system which collects behavioral and clinical data from an annual probability sample of persons in care for HIV infection in the United States. The goals of MMP are to provide nationally representative estimates of clinical (quality of care, access to and use of HIV care, treatment) and behavioral (use of prevention services, medication adherence, and levels of ongoing risk behaviors) outcomes among persons living with HIV infection. To improve the quality and usefulness of data, MMP will increase the representativeness of data compared to legacy systems; will increase the relevance of data for use at the local level (e.g., for Ryan White Comprehensive AIDS Resources Emergency [CARE] and HIV prevention planning groups); and will collect data from people through both interview and medical record review.

This report describes the evolution of supplemental surveillance for behaviors and clinical outcomes, articulates the rationale for the development of this new supplemental HIV surveillance system, briefly describes the MMP methods, and explains how MMP is being used at the local and national levels.

### A Brief History of HIV Behavioral and Clinical Outcomes Surveillance

HIV and AIDS case reporting has been the underpinning of HIV/AIDS surveillance activities since the mid-1980s [1]. All US states have reported AIDS cases using a standard case definition since 1985 [2], and as of 2005, all states conduct surveillance for HIV infection without AIDS [3]. Early in the epidemic, case surveillance data were interpreted in the context of the natural history of HIV infection: clinical disease or severe immunosuppression were predictably (if distantly) related to the time of HIV infection, and AIDS trends accurately reflected past trends in HIV infections [4]. However, as availability and prescription of highly active antiretroviral therapy (HAART) increased, the interval between HIV infection and opportunistic infection (OI) diagnosis or development of severe immunosuppression became highly variable [5]. Thus, case surveillance data on severe immunosuppression and AIDS-defining OI (AIDS-OI) diagnoses were no longer sufficient for monitoring clinical outcomes of HIV infection.

The expansion of the AIDS case definition in 1993 also created challenges for describing the clinical outcomes of HIV infection. Before 1993, ascertainment of diagnoses of AIDS-OIs was quite complete, because diagnosis of an AIDS-OI was a required element for meeting the AIDS case definition. In 1993, the AIDS case definition was expanded to include CD4 count<200 cells/µl as an AIDS-defining criterion [6]. CD4 count<200 cells/µl usually precedes the diagnosis of an AIDS-OI, and most cases are now reported based on this immunologic criterion [7]. For these cases, subsequent AIDS-OI diagnoses are not systematically documented in the case surveillance system. Thus, the completeness of ascertainment of AIDS-OIs decreased considerably after 1993 [7], [8].

In response to the limitations of HIV/AIDS case surveillance to characterize the evolving epidemic, supplemental surveillance systems were developed by the Centers for Disease Control and Prevention (CDC) and state surveillance programs during the 1990s to address emerging data needs. The Adult/Adolescent Spectrum of HIV Disease (ASD) project was implemented in 1990 to collect information on the natural history of HIV/AIDS, and later evolved to include data on treatment and clinical outcomes (e.g., AIDS-OIs, other illnesses, the impact of treatment and prophylaxis) of people with HIV infection who were in care [9]. This facility-based, observational cohort study, which used medical record reviews, operated in 11 US cities from 1990–2004, and observed over 61,000 people in care for HIV infection. ASD data were initially utilized to help determine allocation of resources and to track clinical outcomes; later, to replace case surveillance as the primary means to monitor trends in OIs; and later still, to monitor treatment, survival, and outcomes in the HAART era [10]–[18].

Similarly, the Supplement to HIV/AIDS Surveillance (SHAS) project was implemented to collect behavioral information by interview of people living with HIV infection. SHAS interviewed people living with HIV infection from 1990 to 2004 in 19 states and local areas, providing important information on HIV testing and care-seeking behaviors, access to health care and ongoing sex and drug use behaviors [19]. SHAS data have been used to inform local planning processes and national reporting of behavioral trends among person with HIV infection [20]–[27].

While ASD and SHAS each provided information useful for understanding the epidemic in its various stages, limitations, such as the lack of linked medical record and interview data, limited number of areas participating, and lack of nationally representative estimates for HIV-infected patients in care, resulted in the need for new systems to collect data on behaviors and clinical outcomes [28].

### The Need for a New, Population-Based Surveillance Approach

A new surveillance approach was needed to understand the clinical outcomes and behaviors of people in care for HIV infection because of 3 main factors. First, the introduction of HAART created new challenges for clinical outcomes surveillance. Second, the HIV epidemic now severely impacts more geographic areas in the US, and many people with HIV receive care outside of the major cities where the epidemic–and supplemental surveillance efforts–was centered in earlier years. Third, there are increased needs for representative data to describe the epidemic and related resource needs for care and treatment at the local and national levels.

#### The introduction of HAART and related challenges

Effective administration of HAART to persons living with HIV infection delays the progression of immunosuppression and is associated with decreased incidence of AIDS-OIs and death [29]. This development, after 1996, had significant implications for HIV clinical outcomes surveillance. First, for those persons in care, understanding the extent to which HAART is prescribed as indicated in a variety of practice settings is critical to evaluating our efforts to decrease the severity of HIV disease and to identifying opportunities for improving clinical care and preventing morbidity. Second, the understanding that very high levels of adherence to HAART are required for acceptable suppression of viral load makes the collection of correlated data from medical records and interview a high priority to understand the acceptance of and adherence to recommended antiretroviral therapies. Third, following the availability of HAART, a dichotomy in clinical outcomes for HIV infection has emerged: many persons with HIV infection are living longer, resulting in a strain on available treatment and care resources, while those who do not have timely diagnosis of HIV continue to learn of their HIV infection status only when they are clinically ill or late in the course of infection [30]–[33]. It was important that MMP address multiple subpopulations of people living with HIV infection, including those receiving regular care and those who access care intermittently. Finally, as the number of people living with HIV infection grows, CDC, the Health Resources Services and Administration (HRSA), and the Infectious Disease Society of America have published guidelines for the provision of prevention services to HIV-positive people [34]. There is a need to monitor the provision of these services, and trends in transmission risk behaviors of people in care for HIV infection.

#### Increased geographic heterogeneity of the HIV epidemic

There has been a great increase in the geographic distribution of HIV-infected persons [35], [36]. Previous supplemental surveillance projects were conducted mainly in large metropolitan areas, which were most heavily impacted in the first decade of the epidemic. There are two impacts of this diffusion of HIV morbidity on surveillance requirements. First, it is important to understand the clinical outcomes, quality of care, and related behaviors in diverse settings where HIV care occurs. Quality of care, and clinical outcomes, are related to physician experience in HIV care [37]; in areas with lower morbidity and fewer specialty practices, physician experience and other important factors may vary. Second, more states have substantial morbidity and need data on illnesses and behaviors of HIV-infected persons for local planning and resource allocation for both prevention and care. In 1991, when the Ryan White Comprehensive AIDS Resource Emergency (CARE) Act was first authorized and ASD and SHAS began, there were 16 Eligible Metropolitan Areas (EMAs); in 2006, there were 51 EMAs [38]. Given the substantial expenditure of federal and state funds on HIV care [39] and prevention, all areas receiving Ryan White CARE Act support should have access to the highest quality data for allocation and prioritization purposes.

#### Increased needs for representative data to describe the epidemic and resource needs, and to provide context for existing observational data sources

**Figure 1:**
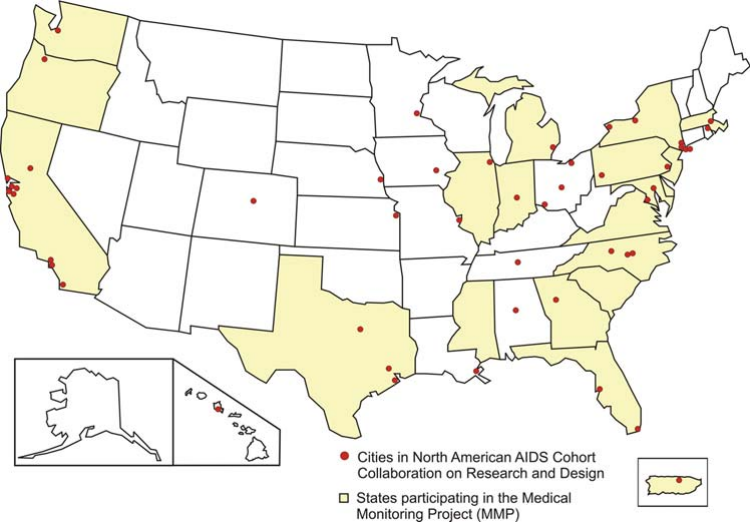
Geographic distribution of US HIV clinical cohorts and Medical Monitoring Project data collection sites, 2007.

There are many existing and previous cohorts that provide data on HIV-infected patients in care in the United States, and these cohorts have made significant contributions to our understanding of the natural history of HIV infection and to treatment recommendations. Recently, progress has been made to combine data from observational cohorts, in order to facilitate more comprehensive datasets and to allow more robust analyses. The North American AIDS Cohort Collaboration on Research and Design (NA-ACCORD) has been established, as part of the International Epidemiologic Databases to Evaluate AIDS (IeDEA) initiative. NA-ACCORD represents over 70,000 HIV-infected patients in care in over 50 clinical sites in the major US cities [40] (Figure 1). MMP will provide a useful complement to NA-ACCORD and individual US clinical cohorts, by providing a nationally representative view of the characteristics of HIV-infected persons in care. MMP data will include patients recruited from a greater diversity of practice settings–including between 800 and 1000 clinical sites each year–and will also include patients in care outside of major metropolitan areas (Figure 1). Having a representative sample of patients in care will provide a frame of reference by which observational cohorts can characterize the inclusion biases in their own populations, and may also be useful to define a reference population, to which data from other cohorts can be standardized in analyses. This may be important in reporting outcomes which are significantly associated with race and ethnicity, because previous CDC-supported studies and many current facility-based cohort studies have racial/ethnic distributions that are not necessarily reflective of the population of adults living with HIV in the US (Table 1).

**Table 1:** Racial/Ethnic Distribution of Adults* Living with HIV/AIDS in the United States, 2003, and in US HIV Cohorts, 2003-2006

Data Source	N	% White	% Black	% Hispanic	% Other
US Case Reports† (2003) [59]	476,749	34	47	17	1
NA-ACCORD overall (range) [40]	71,598 (562-17,125)	46% (10%-65%)	39% (16%-90%)	N/A‡	N/A‡
ASD (2003)	12,477	29	49	20	2
SHAS (2003)	2,371	22	54	20	4

* Aged 13 and older in ASD and the US living HIV/AIDS cases, 18 and older in SHAS

† Estimated number of persons living with HIV/AIDS at the end of 2003 from 33 areas with confidential name-based HIV infection reporting

‡ Not available: data on patients with Hispanic ethnicity are not reported in cohort profile [40]

At the request of Congress, an Institute of Medicine (IOM) committee in 2003 reviewed the status of HIV/AIDS surveillance data and the extent to which data currently collected by the AIDS case surveillance and supplemental surveillance systems were adequate for determining allocation of resources for treatment and care of HIV infection [41]. The IOM committee recommended that HRSA and the CDC evaluate the cost and utility of redesigning studies to assess the specific needs and circumstances of people living with HIV. One of the approaches proposed by the IOM was to coordinate HRSA and CDC efforts to survey a random sample of HIV-infected persons to develop more accurate measures of need for prevention and care services. These recommendations are being met through the implementation of MMP.

### The Medical Monitoring Project: A Population-Based Approach to Behavioral and Clinical Outcomes Surveillance

CDC is working with state and local health departments to obtain a national probability sample of patients in care for HIV infection. The methods were developed in light of an earlier population-based survey of persons in care for HIV infection [42], [43], and earlier CDC pilots of population-based methods [44].

**Figure 2:**
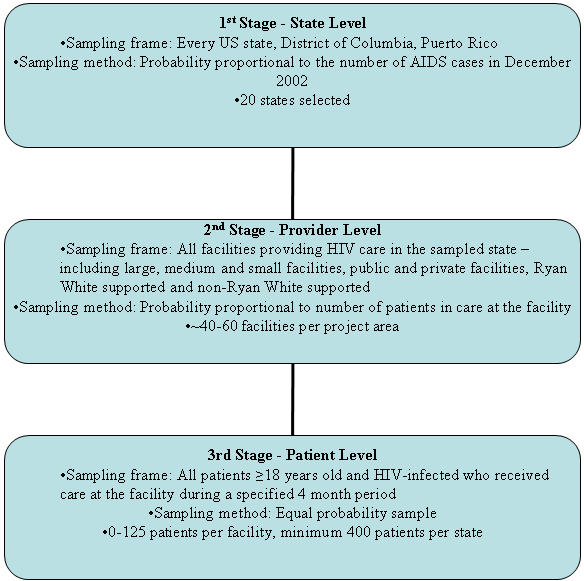
Medical Monitoring Project 3-stage sampling design.

The design is a three-stage sampling approach (Figure 2). The first stage of sampling resulted in the selection of 20 of 52 eligible geographic primary sampling units (PSUs, defined as 50 states, Washington, DC, and Puerto Rico) using probability proportional to size (PPS) sampling methods. Sampling methods ensured representation of all regions of the US (Figure 1). In the second stage, providers of HIV care (i.e., providers that prescribe antiretroviral therapy [ART] or order CD4 or HIV viral load tests) are sampled. The sampling frame of providers is developed in each participating geographic area using data from local HIV/AIDS case surveillance, laboratory reporting, AIDS Drug Assistance Programs and other available data sources. Providers are sampled using PPS methods based on their patient caseload. In the third stage, local HIV/AIDS surveillance staff work with each selected provider to develop a list of HIV-infected patients who received care from the provider at least once in the first four months of the year. From this list, an equal probability sample of patients is chosen [44].

Through an informed consent process, selected patients are offered participation in an interview with the understanding that their medical records will also be reviewed. The types of data collected from the interview and medical record abstraction are represented in Table 2. For most data elements, the interview and medical record abstraction collect data pertaining to the 12 month period prior to the interview date; a few important variables (e.g., prescription of antiretroviral therapy, diagnosis of an AIDS-OI) are documented if they occurred ever following HIV diagnosis.

**Table 2:** Medical Monitoring Project Data Domains, 2007*

Collected by Interview	Collected by Medical Record Abstraction
Demographics	Demographics
Access to health care	Insurance status
Adherence to antiretroviral therapy	AIDS-defining and other illnesses
Unmet need	Laboratory values
Sexual behavior	Antiretroviral and other medications prescribed
Drug and alcohol use history	Substance abuse
	Inpatient, outpatient and emergency room visits

* Complete interview and chart abstraction instruments are available at: http://www.cdc.gov/hiv/topics/treatment/MMP/index.htm

Sampled states have a minimum sample size of 400 patients; for some states with large numbers of prevalent cases, higher sample sizes are allocated (California, 1300; Florida, 800; Illinois, 500; New Jersey, 500; New York, 1200; Pennsylvania, 500; Texas, 800). The minimum sample size will allow the annual description of outcomes of interest–for example, the proportion of eligible patients prescribed prophylaxis for *Pneumocystis jiroveci* pneumonia–with a confidence interval half-width of ± 4 to ± 7% in individual project areas (depending on total sample size in the area), and with a confidence interval half-width of ± 1% in national data.

Data from pilot studies indicate that the differences in results of population-based versus representative samples are meaningful. Estimates from previous surveillance projects demonstrate differences in outcomes reported from population-based versus convenience samples. When comparing the proportion of patients treated according to guidelines in ASD and CDC's Survey of HIV Disease in Care (SHDC) project, the pilot of population-based surveillance methods conducted beginning in 1998, differences were found [44]. In King County, Washington, estimates of proportions of individuals with at least one CD4 or HIV viral load test documented were higher in SHDC patients (95%, 95% CI 91-100) than ASD patients (82%); the opposite was found in Michigan (SHDC 68%, 95% CI 50-86; ASD 87%). This is likely due to differences in the types of providers and facilities that participated in these projects. MMP, being representative of facility types, HIV-infected patient caseloads, and provider experience and specialization will provide more accurate estimates of HIV care and treatment parameters at both the local and national levels.

### Uses of Population-Based Behavioral and Clinical Outcomes Data

#### Local Data Uses

At the local level, MMP data will be useful for local community planning purposes, including the development of local epidemiologic profiles and responding to data requests from agencies which provide resources for HIV care and treatment. MMP will provide information on the characteristics of persons in care for HIV infection and the types of care they are accessing, and will identify needs for prevention and care services among a representative sample of persons in care. Information about access to and use of these services can inform the evaluation of care and prevention services for people living with HIV.

MMP data will allow estimation of unmet need for HIV care and services, and quality of HIV care provided; such estimates are often required by funders of HIV treatment and care [45]. In an effort to reduce the burden on local health jurisdictions and improve comparability of data across reporting areas, HRSA and CDC collaborated on the development of data elements for MMP, and will work together to determine reporting plans that will improve standardization of data collection methods.

A strategy to provide state-level estimates of important behaviors and clinical outcomes using a probability sample will change the quality of information available at the local level in two ways. First, in almost all cases in the past, community planning groups, CARE Act planning consortia and councils have utilized data from projects which, because of recruitment methods, were not necessarily representative of populations living with HIV in the community. Data from a local probability sample would improve the representativeness of the data available to planning groups. Second, data available from past supplemental surveillance projects have not generally been locally interpreted with confidence intervals to reflect the uncertainty around point estimates. MMP will provide planning bodies information about the confidence with which estimates are made. It will be important for CDC and state and local partners to provide training for planning bodies and other users of MMP data to allow appropriate interpretation of the data, and to understand how data available from MMP compare to data from other local projects previously used for local planning processes.

Historically, local areas and their planning bodies have sought separate estimates for population subgroups (e.g., the proportion of persons of color receiving HAART) or to perform statistical testing for differences between subgroups (e.g., to determine if men and women have differences in access to certain services). Although a probability sample approach will allow for estimation of outcomes for such subgroups and significance testing, a limitation of the MMP design is that the numbers of patients represented in some subgroups may be small at the local level. It is not likely that sufficient power will be present for hypothesis testing among subgroups within a project area in any single project year. Samples drawn across successive years can be combined to gain additional statistical power and to analyze data in smaller subgroups of interest.

#### National Data Uses

At the national level, MMP data will be useful for tracking national trends in morbidity; for describing service access and utilization; for focusing and prioritizing national initiatives to improve the provision of treatment and prevention resources; and for benchmarking and evaluating progress towards national prevention and treatment objectives. Annual or bi-annual national estimates of rates of OI diagnoses will be the gold standard for measuring the effectiveness of reducing the severity of HIV-related disease, for describing the characteristics of persons who have progressive HIV disease, and for characterizing the reasons for disease progression. Similarly, a nationally representative sample provides the ideal data source for evaluating progress towards national public health goals, such as describing the proportion of persons receiving appropriate care for HIV infection as described by Healthy People 2010 targets [46]. CDC, HRSA and other governmental agencies are also required to account for use of resources to Congressional funders. For example the Government Performance and Results Act (GPRA) requires reporting of data on prevention of OIs, provision of prevention services, and proportion of CARE Act clients receiving CD4 counts and viral loads [47]. National data should also be useful for documenting the need for treatment resources. Data from the MMP will be of the highest quality for answering national questions about care and treatment needs, the quality of care and treatment, and the use and impact of allocated resources.

Data from the interview portion of the project will also be relevant to evaluation of prevention initiatives for persons living with HIV infection, as described in the Advancing HIV Prevention Initiative and as envisioned in HIV Prevention Strategic Plan goals for reducing the number of people at risk for transmitting HIV infection [48], [49]. Data on key indicators of behavioral risks for transmitting HIV will be available with national, population-based inference, and can be used to determine progress towards national goals and identify populations in need of additional research, improved interventions, or additional funds to support prevention programs.

There are some important limitations to MMP as a surveillance system for clinical outcomes and related behaviors. Because MMP does not have a longitudinal component, data from MMP cannot evaluate outcomes such as survival and effects of therapy over time; data from existing cohorts will be needed to evaluate these outcomes [50]–[53]. The cost per patient recruited and enrolled is greater for MMP than for facility-based studies, and therefore, it is more difficult to obtain adequate sample sizes to make inference to subgroups of interest, especially at the local level.

### Security and Confidentiality

Historically, the legal authority for collecting and reporting data on cases of infectious diseases, including HIV/AIDS, resides with state and local governments [54]. Each state has its own unique legislation, written rules, or regulations mandating the collection of these types of data, and there is considerable variation between jurisdictions [55]. These laws, rules, and regulations allow access to patients' medical records by the health departments for purposes of conducting routine surveillance as part of their mandate to protect the public's health. Names of cases and other unique identifiers collected are retained by state and local jurisdictions and are never sent to CDC. All HIV/AIDS surveillance data have protections at both the state and federal levels. At the state level, state laws and regulations protect surveillance information, limit the uses of data for non-public health purposes, and provide criminal penalties for inappropriate disclosure of surveillance data [56]. At the Federal level, surveillance data are held under the Federal Assurance of Confidentiality, which protects data held by CDC from disclosure for any purpose other than that for which it was collected [57]. In many local areas, legal authority for HIV/AIDS surveillance activities may also extend to the collection of clinical outcomes surveillance data in MMP.

In April 2003, the Health Insurance Portability and Accountability Act (HIPAA) of 1996 was implemented and regulates how covered entities (including most health care delivery organizations) use and disclose certain individually identifiable health information. Surveillance data are specifically exempted from HIPAA because these data are required to be reported to the health department by state and local laws [58]. Health departments conducting MMP are public health authorities, as defined by HIPAA, and many consider MMP to be a surveillance activity. Health care providers may disclose protected health information to public health authorities without individual authorization for the purposes of preventing or controlling disease, for example, as part of HIV/AIDS surveillance activities–including MMP [58].

### Summary

There have been many changes in the HIV epidemic in the US over the past two and a half decades. To address current supplemental surveillance data needs, the reporting requirements for entities providing direct funding for HIV care and prevention services, and recent recommendations from the IOM, CDC and its state and local health department partners have developed a population-based probability sample approach to surveillance for HIV-related behaviors and clinical outcomes, health care utilization, and unmet needs in HIV-infected persons in care. Primary products will be representative state- and national-level estimates of important clinical outcomes of HIV infection, resource utilization, compliance with treatment guidelines, and behavioral outcomes. This approach promises to provide higher quality information for prevention and care planning, resource allocation and evaluation at the national and local levels.
